# A multi-task learning-based network for detecting three-dimensional osteometric landmarks of the shoulder joint

**DOI:** 10.3389/fbioe.2026.1948997

**Published:** 2026-10-28

**Authors:** Wei Han, Fan Li, Cunxiang Ma, Teng Zhang, Jingyu Pan, Xi Chen

**Affiliations:** 1 Department of Orthopaedic Trauma, Beijing Jishuitan Hospital, Capital Medical University, Beijing, China; 2 Beijing Estun Medical Technology Co. Ltd., Beijing, China

**Keywords:** deep learning, landmark detection, multi-task learning, shoulder joint, surgical planning

## Abstract

**Background:**

Mirror-based preoperative planning for total shoulder arthroplasty relies on accurate three-dimensional (3D) localization of bony landmarks on the contralateral, anatomically intact shoulder. Manual annotation is time-consuming and shows inter- and intra-observer variability. The aim of this study was to develop and evaluate a multi-task deep-learning network for automated 3D bony landmark detection on contralateral shoulder CT.

**Methods:**

In this single-center retrospective study, 179 patients with intact contralateral shoulder CT scans were split at the patient level into training (n = 125), validation (n = 18), and test (n = 36) sets. We developed a dual-decoder 3D BiFormer-UNet for humerus and scapula segmentation and heatmap-based localization of 14 anatomical landmarks. A cross-task feature-fusion (CtFF) module transferred bone-structural guidance from the segmentation decoder to the landmark decoder. The network was trained end-to-end using a composite loss comprising hard-negative-mining heatmap regression, a geometric structure constraint, distance-aware similarity learning, and auxiliary segmentation loss. Landmark localization was evaluated using MRE and SDR at thresholds of 2.0, 2.5, 3.0, and 4.0 mm, while segmentation was assessed using the Dice coefficient. Per-case MRE was compared with four 3D baseline models using paired tests with Holm–Bonferroni correction.

**Results:**

In the 36-case test set, the proposed model achieved an MRE of 2.1 ± 1.2 mm (95% CI, 1.7–2.5 mm) and SDRs of 72.0%, 81.8%, 90.3%, and 96.7% at thresholds of 2.0, 2.5, 3.0, and 4.0 mm, respectively. Its MRE was significantly lower than those of 3D U-Net, V-Net, 3D HRNet adaptation, and H3DE-Net (all Holm–Bonferroni-adjusted P < 0.05). On the test set, the auxiliary segmentation branch achieved mean Dice coefficients of 0.83 ± 0.05 for the humerus and 0.78 ± 0.07 for the scapula. Internally defined center landmarks, particularly the humeral head rotation center, anatomical neck center, and proximal and distal medullary canal centers, showed larger localization errors (2.3–2.6 mm) than most surface landmarks.

**Conclusion:**

The proposed multi-task framework demonstrated promising 3D landmark localization performance on intact contralateral shoulder CT by integrating global anatomical context with bone-structural guidance. It may serve as an upstream localization component for mirror-based shoulder arthroplasty planning, although external validation is required before clinical use.

## Introduction

The convergence of medical imaging, computer-assisted techniques, robotics, and artificial intelligence (AI) is transforming joint surgery from qualitative, experience-based practice toward quantitative, image-guided planning (Kong et al., 2024; Li et al., 2024; Bu et al., 2024). In total shoulder arthroplasty (TSA), preoperative three-dimensional (3D) assessment of humeral and scapular anatomy—including glenoid version, glenoid inclination, and humeral head retroversion—informs implant selection and positioning (Gates et al., 2020; Scalise et al., 2008; Werner et al., 2022). Because these measurements depend on the reproducible identification of bony landmarks, accurate landmark localization is a prerequisite for reliable preoperative measurement and planning (Boileau et al., 2018; Satir et al., 2024; Spangenberg et al., 2025).

Although automated 3D measurements have been reported (Boileau et al., 2018; Satir et al., 2024), many clinical workflows still require manual or semi-automated landmark review. Such workflows are time-consuming and operator-dependent, and substantial intersurgeon and intrasurgeon variability in shoulder arthroplasty planning has been documented (Parsons et al., 2020). For shoulders with severe bone loss or deformity, contralateral reconstruction (Giraudon et al., 2024) and statistical shape modeling (Fleet et al., 2024) represent complementary strategies for estimating premorbid glenoid anatomy. In a mirror-based planning workflow, the reliability of subsequent registration and reconstruction therefore depends on stable localization of anatomical landmarks on the anatomically intact contralateral shoulder.

Recent studies have begun to automate shoulder-specific image analysis. Satir et al. combined scapular segmentation with localization of 13 scapular and glenoid landmarks on CT and reported landmark errors of approximately 1–2.5 mm in normal and osteoarthritic shoulders (Satir et al., 2024). Croci et al. developed an nnU-Net-based method to detect and track five shoulder landmarks on fluoroscopic images (Croci et al., 2024), whereas Spangenberg et al. predicted the humeral resection plane from CT-derived three-dimensional humeral models for shoulder arthroplasty planning (Spangenberg et al., 2025). At the workflow level, a real-world evaluation of commercial shoulder arthroplasty software showed that auto-planning reduced planning time and the number of user actions (Mahe et al., 2026). In parallel, recent three-dimensional medical landmark methods have explored coarse-to-fine (Cui et al., 2025) and super-resolution heatmap frameworks (Zhang et al., 2024), while transformer-based volumetric architectures such as UNETR have demonstrated the value of long-range contextual modeling in three-dimensional medical images (Hatamizadeh et al., 2022). Torosdagli et al. developed a multi-stage framework that first segmented the mandible from CBCT images and subsequently localized anatomical landmarks using geodesic representations derived from the segmented bone anatomy (Torosdagli et al., 2019). Their study showed the feasibility of using segmentation-derived anatomical structure to provide spatial guidance for landmark localization. However, simultaneous localization of humeral and scapular landmarks on contralateral shoulder CT using an end-to-end, segmentation-guided multi-task framework remains insufficiently studied.

Automated landmark detection in shoulder CT remains challenging for four reasons. First, the scapula has a complex, individually variable curvature, and the proximal humerus assumes diverse postures with respect to abduction and rotation, conventional convolutional architectures may have limited effective receptive fields for modeling long-range anatomical relationships. Second, high-quality 3D shoulder CT datasets are limited and manual annotations carry observer-dependent noise, encouraging over-fitting. Third, most existing approaches treat segmentation and landmark detection independently and do not exploit bone-shape priors that segmentation can provide. Fourth, conventional heatmap-regression losses treat easy background voxels and rare hard negatives equally and rarely model inter-landmark geometric relations or feature-space similarity explicitly.

Building on the general principle of segmentation-derived structural guidance, we developed an end-to-end dual-decoder framework with task-specific cross-task feature fusion between the segmentation and landmark-detection branches. The scope of the present study was deliberately limited to the contralateral, anatomically intact shoulder, with the goal of supporting downstream mirror registration and reconstruction of the affected side. We hypothesized that, on contralateral shoulder CT, jointly modeling long-range anatomical dependencies and bone-surface structural priors would yield more accurate 3D landmark localization than conventional 3D convolutional and high-resolution baseline networks and improve successful detection rates at predefined distance thresholds.

## Methods

### Study design and ethical approval

This single-center, retrospective, methodological validation study was approved by the Institutional Review Board of Beijing Jishuitan Hospital, Capital Medical University (Jilun [L2025] No. [014]-00). The requirement for individual informed consent was waived by the ethics committee given the retrospective nature of the study and the use of de-identified imaging data; however, all patients had previously signed the hospital’s general informed consent form covering the use of clinical data for research purposes. The study was conducted in accordance with the Declaration of Helsinki and complied with applicable institutional data governance policies.

### Patient selection and dataset

To validate the ability of the method described in this paper to automatically localize anatomical landmarks on the unaffected side during the preoperative planning process for mirror surgery, this study retrospectively collected CT scan data of the shoulder joint from 179 patients at Beijing Jishuitan Hospital, Capital Medical University. The underlying conditions affecting the affected shoulder included shoulder osteoarthritis, proximal humeral fractures, and shoulder dislocations; however, only CT images of the anatomically intact contralateral shoulder were included in the analysis. It is important to emphasize that this study targets the healthy/intact side of the shoulder joint in the context of mirror-based preoperative planning. Its objective is to provide a stable upstream anatomical reference for subsequent mirror registration and reconstruction of the affected side; therefore, the generalizability of direct detection on abnormally deformed or pathological bone surfaces is not a primary validation endpoint of this study.

The 179 patients were randomly split at the patient level into training (n = 125; 70%), validation (n = 18; 10%), and test (n = 36; 20%) sets. All CT data from the same patient were assigned exclusively to one subset to prevent data leakage.

### Anatomical landmark definitions

Fourteen bony landmarks were annotated for each shoulder CT scan. Table 1 provides the complete list with anatomical descriptions, coordinate definitions, and clinical relevance.

**TABLE 1:** Definitions of the 14 anatomical landmarks annotated on shoulder CT.

#	Landmark	Anatomical definition	Coordinate type	Clinical relevance
1	Humeral head rotation center (HRC)	Center of the best-fit humeral-head sphere	Sphere-fitted center	Humeral head center, version, and offset measurement
2	Anatomical neck center (ANC)	Center of the anatomical neck cross-section	Circle-fitted center	Anatomical-neck and head–neck alignment References
3	Medial epicondyle (ME)	Most medial point of the medial epicondyle	Surface point	Transepicondylar-axis and rotational-alignment References
4	Lateral epicondyle (LE)	Most lateral point of the lateral epicondyle	Surface point	Transepicondylar-axis and rotational-alignment References
5	Proximal medullary canal center (PMC)	Proximal humeral medullary-canal center	Cross-sectional center	Humeral shaft-axis definition
6	Distal medullary canal center (DMC)	Distal humeral medullary-canal center	Cross-sectional center	Humeral shaft-axis definition
7	Greater tubercle (GT)	Most prominent point of the greater tubercle	Surface point	Tuberosity positioning and humeral rotational alignment
8	Glenoid deepest point (GDP)	Deepest point of the glenoid fossa	Surface point	Glenoid-surface References for version and inclination measurement
9	Superior glenoid margin (SGM)	Most superior point of the glenoid rim	Surface point	Superior–inferior glenoid-axis References
10	Trigonum spinae (TS)	Medial endpoint of the scapular spine	Surface point	Friedman-line and scapular-axis References
11	Angulus inferior (AI)	Most inferior point of the scapula	Surface point	Scapular coordinate-system References
12	Inferior glenoid margin (IGM)	Most inferior point of the glenoid rim	Surface point	Superior–inferior glenoid-axis References
13	Anterior glenoid margin (AGM)	Most anterior point of the glenoid rim	Surface point	Glenoid version and anterior bone-stock assessment
14	Posterior glenoid margin (PGM)	Most posterior point of the glenoid rim	Surface point	Glenoid version and posterior bone-stock assessment

The HRC was defined as the center of the best-fit sphere fitted to the humeral head articular surface. For the ANC, eight points sampled around the anatomical-neck margin of the humeral head were used to fit a three-dimensional circle, whose center was taken as the ANC. The PMC and DMC were defined as the centers of the medullary canal on cross-sectional planes through the proximal humeral shaft distal to the humeral head and the distal humeral shaft proximal to the humeral condyles, respectively. Because these landmarks were used primarily to define the humeral shaft axis for coordinate-system construction, their exact proximodistal positions were not fixed; limited longitudinal variation was permitted, provided that both points remained centered within the medullary canal.

### Ground-truth annotation

All CT images were stored in standard DICOM format and annotated using 3D Slicer. Two orthopedic surgeons with at least 3 years of clinical experience independently performed slice-by-slice segmentation of the humerus and scapula and manually identified the 14 anatomical landmarks listed in Table 1. Disagreements were adjudicated by a senior orthopedic surgeon with more than 15 years of experience, producing the final consensus segmentation masks and landmark coordinates used as ground truth.

Annotation reliability was evaluated in a random subset of 20 cases. Both annotators repeated the annotations after a 2-week washout period. Interobserver and intraobserver reliability were quantified using the intraclass correlation coefficient (ICC; two-way mixed-effects, single-measure, absolute-agreement model) and the mean Euclidean distance between corresponding landmarks. Across all landmarks, the interobserver ICC was 0.94 (95% CI, 0.91–0.96), with a mean distance of 0.8 ± 0.5 mm. The intraobserver ICC was 0.96 (95% CI, 0.93–0.97), with a mean repeatability distance of 0.5 ± 0.3 mm. These results indicate high annotation reliability.

### Data representation

This paper uses 3D shoulder joint CT data as its subject of study, and denotes the data from a single subject as $I\in R^{1\times D\times H\times W}$ , where D , H , W represents the number of voxels in the axial, coronal, and sagittal planes, respectively. The corresponding bone structure segmentation labels are denoted as $M\in {\left\{0,1,...,C\right\}}^{D\times H\times W}$ , C represents the number of foreground classes. The set of bony landmarks of the shoulder joint is denoted as $P=\left\{p_{k}\in R^{3}|k=1,...,K\right\}$ , K indicates the number of landmarks, $p_{k}=\left(z_{k},y_{k},x_{k}\right)$ represents the position of the k th landmark in the voxel coordinate system.

In addition, to adapt to the heatmap regression model, this paper converts discrete coordinate annotations into a multi-channel 3D Gaussian heatmap representation. For each anatomical landmark $p_{k}$ , the corresponding heatmap is generated using a 3D Gaussian function (Equation 1):

$$H_{k}^{gt}\left(z,y,x\right)=\exp\left(-\frac{{\left\|\left[z,y,x\right]-p_{k}\right\|}_{2}^{2}}{2\sigma^{2}}\right)$$ (1)

The σ parameter controls the peak spread; the Gaussian kernel radius was set to 2 voxels. The heat map of all marker points is stacked as follows: $H^{gt}\in {\left[0,1\right]}^{K\times D\times H\times W}$ . The goal of the model’s landmark prediction branch is to predict $H^{pred}\approx H^{gt}$ , identify landmark coordinates by finding the probability peaks in each channel during the inference stage.

### Overall network architecture

As shown in Figure 1, the network model proposed in this paper consists primarily of three components: (1) a BiFormer-based shared feature extraction module, used to capture long-range dependencies and global contextual information in 3D CT images; (2) a dual-branch decoder structure, which outputs probability maps for humerus and scapula segmentation and keypoint heatmaps, respectively; (3) a CtFF, which directs the dual-channel bony structure attention generated by the segmentation branch to the detection branch.

**FIGURE 1:**
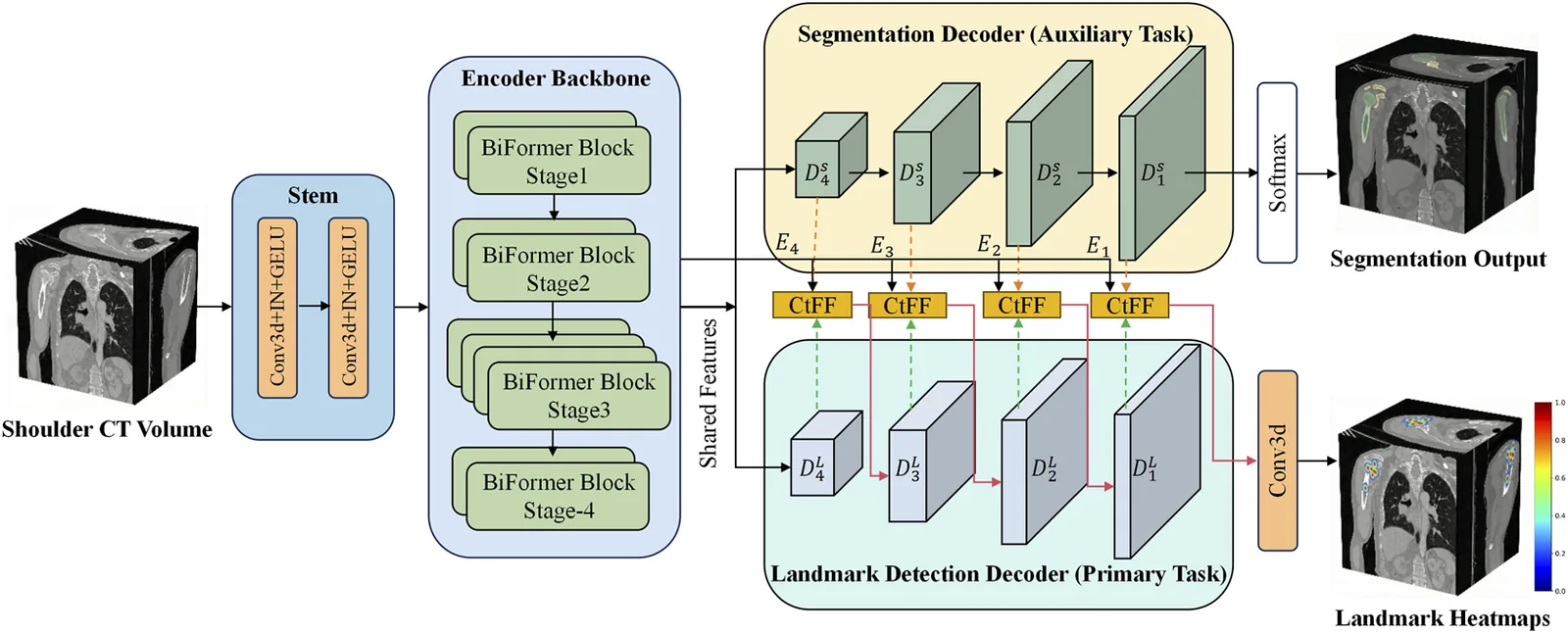
Overall architecture of the proposed multi-task dual-decoder network. The BiFormer-based encoder extracts multi-scale features from the input CT volume. The auxiliary segmentation decoder predicts humerus and scapula masks, while the landmark heatmap decoder (enhanced by CtFF modules) predicts 14 anatomical landmarks.

The model input consists of preprocessed 3D shoulder joint CT image tensors I . Firstly, the features pass through two feature extraction modules—each consisting of Conv3d, InstanceNorm, and GELU—to extract low-level edge and texture features and perform preliminary scale compression. The features then enter the BiFormer encoder, which consists of four stages that progressively reduce spatial resolution and increase the number of channels from top to bottom. The multiscale features output from each stage are simultaneously fed into two decoder branches: the auxiliary segmentation branch jointly predicts probability maps for three classes—background, humerus, and scapula—at the voxel level, with the humerus and scapula foreground channels providing explicit morphological priors for the main task; the detection branch outputs a probability heatmap of keypoints after fusing structural features. During training, network parameters are optimized end-to-end using a multi-task joint loss.

The total number of trainable parameters was approximately 29.3 million. All normalization layers used Instance Normalization. The BiFormer encoder stages used region sizes of R = 4 and routing parameter k as specified in Table 2.

**TABLE 2:** Detailed architecture configuration of the proposed network.

Component	Depth (blocks)	Channels	Region size (R^3^)	Routing k	Heads	MLP hidden dimension
Stage 1	4	64	4 × 4 × 4	2	1	32
Stage 2	4	128	4 × 4 × 4	4	2	64
Stage 3	6	256	4 × 4 × 4	4	4	128
Stage 4	4	512	4 × 4 × 4	4	8	256
Seg. Decoder L1–L4	—	64/128/256/512	—	—	—	—
Det. Decoder L1–L4	—	64/128/256/512	—	—	—	—
CtFF modules	—	64/128/256/512	—	—	—	—

### BiFormer-based feature encoder

Accurate localization of key points in the shoulder joint depends not only on local texture features (such as the edges of bony prominences) but also on global anatomical relationships (such as the spatial relative position of the humeral head and the glenoid cavity). To effectively establish this long-range spatial dependency in 3D body data while avoiding the significant computational overhead associated with traditional full-attention mechanisms, this paper proposes a 3D feature encoder based on BiFormer (Zhu et al., 2023).

The core component of the encoder is the 3D BRA mechanism, which achieves a balance between computational efficiency and global receptive fields through a dynamic sparsification strategy. As shown in Figure 2, we extended the original 2D BRA to 3D space to accommodate the volumetric characteristics of orthopedic CT images. The computational process primarily consists of three steps: region segmentation, coarse-grained routing, and fine-grained attention calculation (Equation 2):

1. 3D Region Partitioning and Mapping. Given an input 3D feature map: *F* $\in R^{C\times D\times H\times W}$ , we first partition it into h×w×d non-overlapping local cubic regions, each containing HWD/hwd feature vectors. Through a linear mapping, we obtain the Query, Key, and Value tensors: $Q,K,V\in R^{C\times D\times H\times W}$ .
2. Region-level routing and semantic aggregation. To filter out interference from irrelevant background (such as soft tissue), a coarse-grained screening is first performed at the region level. The average feature vector for each region is computed to obtain region-level queries $Q^{r}$ and region-level keys $K^{r}$ . The inter-region adjacency matrix $A^{r}=Q^{r}{\left(K^{r}\right)}^{T}$ is calculated to measure the semantic relevance between different anatomical regions. Subsequently, for each query region, the $A^{r}$ most relevant target regions are retained using the adjacency matrix k . Based on the routing index, key-value pairs from these k relevant regions are collected through aggregation operations to construct targeted key-value tensors $K^{g}$ and $V^{g}$ . This process effectively eliminates redundant background information, allowing computations to focus on regions with clear anatomical associations.
3. Fine-grained attention computation. After completing the route aggregation, we compute token attention within the retained local context. For each voxel feature in the region, we perform multi-head self-attention using its own query vector Q and the aggregated key-value pairs $K^{g}$ and $V^{g}$ :

$$\begin{array}{l} O=Attention\left(Q,K^{g},V^{g}\right)\\ =Softmax\left(\frac{Q{\left(K^{g}\right)}^{T}}{\sqrt{d_{k}}}\right)V^{g}+LCE\left(V\right) \end{array}$$ (2)

**FIGURE 2:**
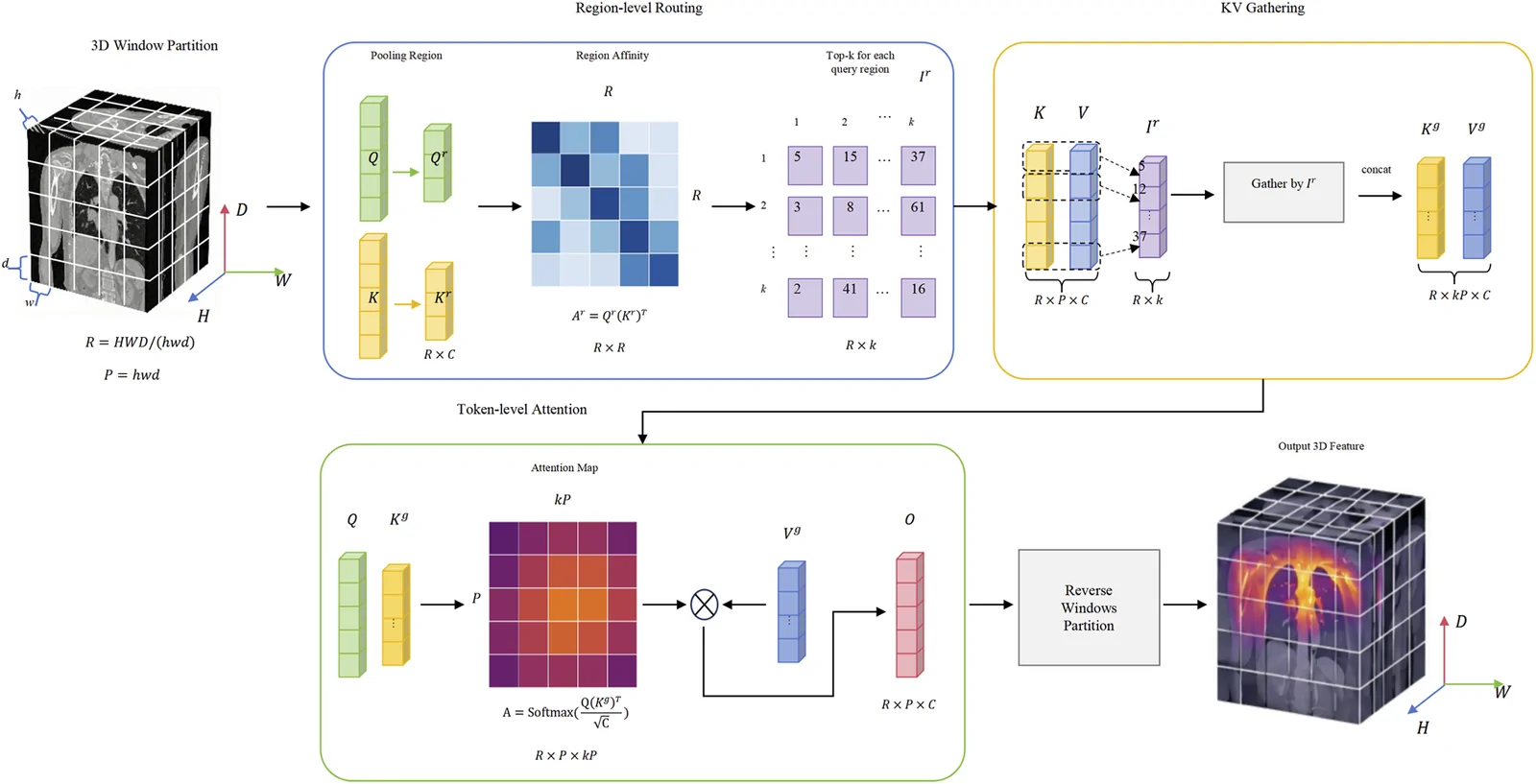
Computational process of the 3D bi-level routing attention (BRA) mechanism. (1) 3D region partitioning; (2) region-level routing and semantic aggregation; (3) fine-grained attention computation within selected regions.

Here, $LCE\left(\cdot \right)$ denotes the local context enhancement term generated by deep convolutions. Ultimately, this mechanism fuses the 3D feature tensor representing the global anatomical context O . This design enables the encoder to capture long-range dependencies across the entire shoulder joint while processing CT data, while also keeping computational complexity within an acceptable range.

To fully integrate local feature details with global semantic information, we embed the aforementioned 3D bi-level routing attention mechanism into the feature extraction module to construct the BiFormer Block. As shown in Figure 3, this module effectively integrates deep convolutions with a sparse attention mechanism through a special residual connection design. A complete BiFormer Block consists of the following three substructures, which are executed sequentially:

1. Residual Deep Convolution (Residual DWConv): The input features first pass through a 5×5×5 DWConv with residual connections. This layer captures local high-frequency information in CT images (such as bone texture and edges) and provides implicit spatial encoding for the subsequent Transformer architecture. The residual connections ensure effective gradient propagation.
2. BRA: The features processed by the DWConv layer first undergo Layer Normalization (LN) and are then fed into the BRA module. This module utilizes the sparse routing mechanism described above to efficiently capture long-range anatomical dependencies in 3D space and fuses them with the original features via residual connections.
3. Multilayer Perceptron (MLP): The features fused with global attention undergo another layer of LN, then enter the MLP for channel-dimension feature transformation and nonlinear mapping, and are finally output as the final features via a residual connection.

**FIGURE 3:**
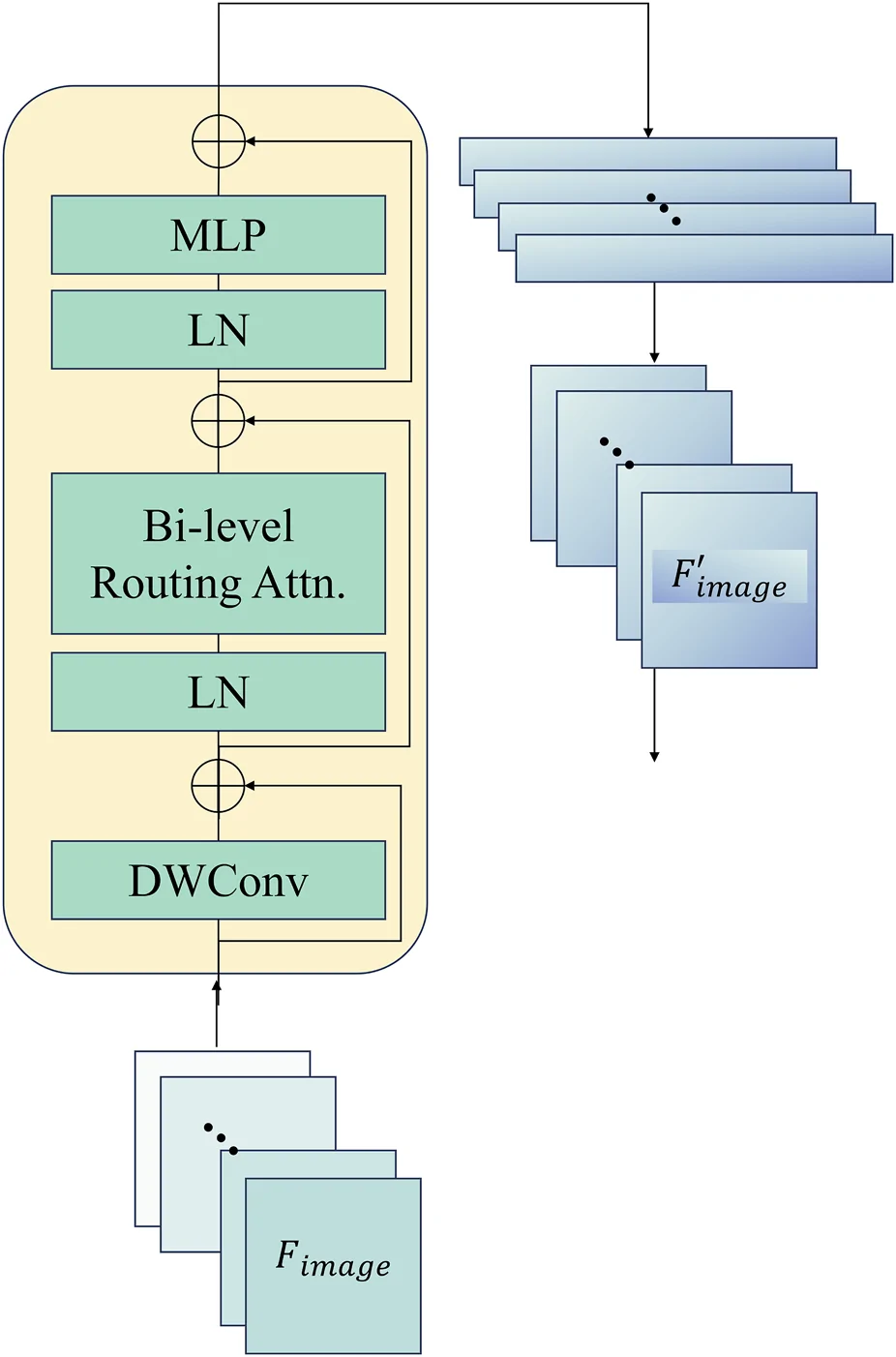
Structure of the BiFormer Block. The block consists of three sequential substructures: residual deep convolution (Residual DWConv), bi-level routing attention (BRA), and multi-layer perceptron (MLP), connected via residual connections.

By stacking modules of this structure, the encoder can progressively extract hierarchical features—ranging from local anatomical details to global skeletal topology—layer by layer, providing a solid feature foundation for subsequent dual-task decoding.

### Dual decoder with cross-task feature fusion

The segmentation decoder produced voxel-wise probability maps for background, humerus, and scapula. The two foreground channels were used as structural guidance for the landmark decoder. The landmark decoder shared the same upsampling topology and integrated CtFF modules at corresponding levels. At each level, CtFF (Figure 4) concatenated the segmentation and detection features along the channel dimension, generated a dual-channel spatial attention map that highlighted the humerus and scapula, used this map to gate the detection feature, and finally concatenated the gated feature with the same-scale encoder feature to recover fine-grained texture.

**FIGURE 4:**
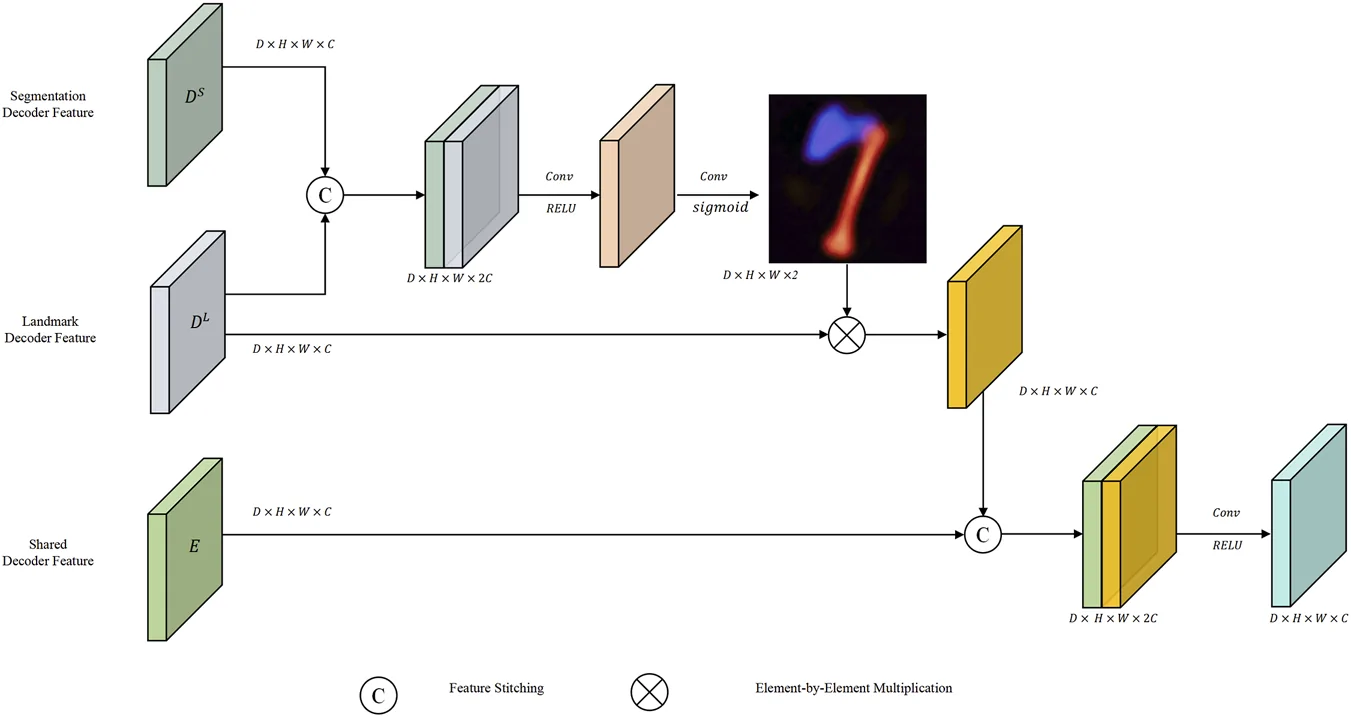
Structure of the cross-task feature-fusion (CtFF) module. Segmentation features and detection features are concatenated along the channel dimension to generate a dual-channel spatial attention map, which gates the detection features before concatenation with encoder skip-connection features.

### Training objective

To further enhance the model’s robustness in complex scenes, we designed a composite loss function $L_{total}$ , consisting of two components: a landmark detection loss and an auxiliary segmentation loss (Table 3). The segmentation sub-task employs a weighted combination of Dice loss and cross-entropy (CE) loss to address the class imbalance between foreground skeletons and background voxels (Equation 3):

$$L_{seg}=L_{Dice}+L_{CE}$$ (3)

**TABLE 3:** Loss function hyperparameters and their selection rationale.

Loss component	Symbol	Weight	Selection basis
Hard negative mining	1 (fixed)	1.0	Baseline References
Geometric constraint	α	0.15	Validation-set preliminary experiments
Similarity learning	β	0.2	Validation-set preliminary experiments
Segmentation	γ	1.0	Validation-set preliminary experiments
τ_1_ (near margin)	—	0.3	Preliminary experiments
τ_2_ (far margin)	—	0.2	Preliminary experiments

In particular, $L_{Dice}$ focuses on the overlap of regions to ensure overall consistency in the skeletal contours; $L_{CE}$ focuses on the accuracy of voxel classification.

The loss function for the landmark detection main task consists of three components: hard negative mining loss, geometric structure constraint loss, and distance-aware similarity learning loss.

1. Hard Negative Mining Loss ( $L_{NHM}$ ). In heatmap regression, the vast majority of voxels are background (negative samples), which can easily dominate the backpropagation of gradients. Furthermore, bone spurs around the shoulder joint or the edges of adjacent bones often produce features similar to keypoints, forming “hard negative samples” (Lin et al., 2017). To address this, we modified the Mean Square Error loss by introducing a mechanism for dynamically adjusting weights (Equation 4):

$$L_{NHM}=\sum_{k=1}^{N}\sum_{p\in \Omega }w\left(p\right)\cdot {\left\|H_{k}\left(p\right)-{\hat{H}}_{k}\left(p\right)\right\|}^{2}$$ (4)

Here, $H_{k}\left(p\right)$ and ${\hat{H}}_{k}\left(p\right)$ represent the predicted heatmap value and the true value, respectively, for the k th key point at position p . The weight $w\left(p\right)$ is defined such that a higher weight is assigned when the position is in the background and the prediction error is large, forcing the network to focus on distinguishing true anatomical landmarks from easily confused bony structures.

2. Geometric Structure Constraint Loss ( $L_{geo}$ ) There are inherent topological relationships between anatomical landmarks of the shoulder joint. To leverage this prior knowledge, this paper proposes a geometric constraint loss. Let $P_{i}$ and $P_{j}$ denote the 3D coordinates of two key points; the difference between the Euclidean distance $d_{pred}^{ij}$ predicted by the network and the true anatomical distance $d_{gt}^{ij}$ should be minimized (Equation 5):

$$L_{geo}=\sum_{\left(i,j\right)\in \varepsilon }{\left\|d_{pred}^{ij}-d_{gt}^{ij}\right\|}_{1}$$ (5)

The set of edges connecting keypoints marked with ε is defined based on anatomical criteria. This loss function constrains the spatial distribution of keypoints, preventing anatomically implausible landmark configurations.

3. Distance-Aware Similarity Learning Loss ( $L_{sim}$ ) To enhance the discriminative power of features, we introduce metric learning into the feature space. This loss function aims to bring the feature representations of keypoints closer to those of their surrounding neighborhoods, while simultaneously increasing the feature distance between keypoints and background regions (Equation 6).

$$L_{sim}=\sum_{k=1}^{K}\begin{array}{l} \max\left(0,\Delta_{pos}-S\left(f_{k},f_{pos}\right)\right)+\\ \max\left(0,S\left(f_{i},f_{neg}\right)-\Delta_{neg}\right) \end{array}$$ (6)

Here, $S\left(\cdot \right)$ denotes cosine similarity, $f_{i}$ represents the keypoint feature vector, $f_{pos}$ represents the keypoint neighborhood features, and $f_{neg}$ represents the background features. $\Delta_{pos}$ and $\Delta_{neg}$ are the set thresholds. This loss function encourages the network to learn keypoint features with high discriminative power.

The total loss for the final network is defined as (Equation 7):

$$L_{total}=L_{HNM}+\alpha L_{geo}+\beta L_{sim}+\gamma L_{seg}$$ (7)

In this context, α , β , and γ are hyperparameters used to balance the weights of each task. Through joint optimization, the network is able to accurately segment skeletal structures while achieving high-precision landmark detection.

### Preprocessing and training details

All CT volumes were resampled to an isotropic voxel spacing of 1.0 mm. HU values were clipped to [−200, 1,500] and linearly normalized to [0, 1]. The resampled volumes were then resized to 192 × 192 × 192 voxels for network input. The same spatial transformations were applied to the segmentation masks and landmark coordinates. During inference, the predicted coordinates were mapped back to the original physical coordinate system. The implementation and training settings are summarized in Table 4.

**TABLE 4:** Training hyperparameters and implementation details.

Parameter	Value
Input dimensions	192 × 192 × 192
Optimizer	AdamW
Initial learning rate	0.0003
LR schedule	Cosine annealing
Maximum number of epochs	200
Early stopping	Patience = 40 epochs on validation MRE; minimum delta = 0.01 mm
Batch size	6
Gradient clipping	Max norm = 5.0
Random seed	42
Hardware	2 × NVIDIA GeForce RTX 4090 (24 GB each)
Framework	PyTorch 2.5.1, CUDA 12.9, cuDNN 9.x
Validation monitoring	MRE on validation set, evaluated every 5 epochs
Gaussian kernel radius	2 voxels
Soft-argmax temperature (τ)	1.0

During training, spatial augmentations were applied jointly to the CT volumes, segmentation masks, and landmark coordinates. These included random rotations of ±10° along each axis (probability, 0.5), isotropic scaling by a factor of 0.9–1.1 (probability, 0.3), and elastic deformation with a control-point spacing of 20 voxels and a maximum displacement of 5 voxels (probability, 0.2).

The heatmap output by the network represents the probability distribution of keypoint locations. To obtain sub-voxel landmark coordinates while preserving differentiability, we used the local soft-argmax algorithm instead of the conventional argmax operation. For the prediction heatmap $H_{k}$ of the k th keypoint, we first use max-pooling to find the peak’s coarse coordinates $p_{\max}$ , and then calculate the expected coordinates within a local neighborhood $p_{\max}$ centered at Ω (Equation 8):

$$P_{k}=\sum_{p\in \Omega }p\cdot \frac{\exp\left(\tau H_{k}\left(p\right)\right)}{\sum_{q\in \Omega }\exp\left(\tau H_{k}\left(q\right)\right)}$$ (8)

Here, τ represents the temperature coefficient, which is used to control the sharpness of the distribution. This method effectively utilizes the information surrounding the peak to improve the accuracy of coordinate regression.

### Baseline implementation and fairness

We compared the proposed method with four three-dimensional baseline networks: 3D U-Net (Çiçek et al., 2016), V-Net (Milletari et al., 2016), a 3D adaptation of HRNet (Wang et al., 2021), and H3DE-Net (Hu et al., 2025). All models used the same patient-level data split, preprocessing pipeline, input dimensions, data augmentation, optimizer, learning-rate schedule, maximum number of epochs, checkpoint-selection criterion, Gaussian heatmap formulation, and local soft-argmax procedure. These standardized settings minimized differences unrelated to model design.

The comparison networks were optimized using the conventional voxel-wise mean squared error (MSE) loss between the predicted and ground-truth Gaussian heatmaps, whereas the proposed network was trained using the proposed loss terms ( $L_{NHM}$ , $L_{geo}$ , $L_{sim}$ ). Therefore, the comparison evaluated the complete implementation of each method, while the individual contributions of the proposed loss terms were examined separately through ablation experiments. All baseline models were configured to output 14 landmark heatmaps. For 3D U-Net and V-Net, the original segmentation heads were replaced with 14-channel heatmap-regression heads. HRNet was extended to three dimensions by replacing its two-dimensional convolution, pooling, upsampling, and feature-fusion operations with their three-dimensional counterparts, while retaining the original parallel multi-resolution architecture and repeated feature-fusion topology (Wang et al., 2021). Its final prediction layer was implemented as a 1 × 1 × 1 convolution with 14 output channels. H3DE-Net was implemented following its original architecture (Hu et al., 2025), with the final prediction head adjusted to output 14 landmark heatmaps.

### Outcome measures

Landmark localization performance was evaluated using mean radial error (MRE) and successful detection rate (SDR), which are commonly used metrics in medical landmark detection studies (Cui et al., 2025; Zhang et al., 2024; Hu et al., 2025; Wang et al., 2015). MRE quantifies the mean Euclidean distance between the predicted and ground-truth landmark coordinates and therefore directly reflects localization accuracy. It is calculated as follows Equation 9:

$$MRE=\frac{1}{N\times K}\sum_{i=1}^{N}\sum_{k=1}^{K}{\left\|P_{i,k}-G_{i,k}\right\|}_{2}$$ (9)

Here, N denotes the number of samples in the test set, and K denotes the number of landmark points per sample (in this study, K=14 ). $P_{i,k}$ and $G_{i,k}$ represent the predicted coordinates and true coordinates of the k th landmark point in the i th sample, respectively. ${\left\|\cdot \right\|}_{2}$ denotes the $L_{2}$ -norm. A smaller MRE value indicates higher localization accuracy of the model.

SDR is used to evaluate a model’s robustness under specific accuracy requirements and is defined as the percentage of landmark points with prediction errors below a given threshold out of the total number of landmark points. To characterize localization performance across different levels of spatial accuracy, SDR was evaluated at four prespecified thresholds: 2.0, 2.5, 3.0, and 4.0 mm. The formula for calculating SDR is defined as Equation 10:

$$SDR_{\delta }=\frac{1}{N\times K}\sum_{i=1}^{N}\sum_{k=1}^{K}\chi_{A}\left({\left\|P_{i,k}-G_{i,k}\right\|}_{2}<\delta \right)\times 100\%$$ (10)

In this context, $\chi_{A}\left(\cdot \right)$ denotes an indicator function that takes the value 1 when the condition is met and 0 otherwise. $\delta \in \left\{2mm,2.5mm,3mm,4mm\right\}$ . A higher SDR indicates more reliable landmark localization at the specified distance threshold.

Per-case MRE values were compared between the proposed method and each baseline model. For each comparison, the normality of the within-case MRE differences (baseline minus proposed) was assessed using the Shapiro–Wilk test and Q–Q plots. Because no substantial departures from normality were identified, paired t-tests were used for the primary comparisons. Wilcoxon signed-rank tests were additionally performed as sensitivity analyses. Within each family of four model comparisons, P values were adjusted using the Holm–Bonferroni method. Ninety-five percent confidence intervals for model-level MRE, SDR, landmark-specific mean errors, and paired mean MRE differences were estimated using 10,000 patient-level bootstrap resamples with the percentile method. All tests were two-sided, and an adjusted P value <0.05 was considered statistically significant.

## Results

To evaluate comparative landmark-localization performance, the proposed method was compared with the four baseline networks specified in the Methods section. Table 5 summarizes the landmark-localization performance of the proposed method and four baseline networks on the test set. The proposed method achieved the lowest MRE (2.1 ± 1.2 mm) and the highest SDR at all evaluated thresholds. Compared with H3DE-Net, the MRE decreased from 2.6 ± 1.5 mm to 2.1 ± 1.2 mm, while SDR@2.0 mm increased from 64.3% to 72.0%.

**TABLE 5:** Comparison of landmark-localization performance on the test set (n = 36). (*Adjusted P < 0.05 versus the proposed method based on paired t-tests with Holm–Bonferroni correction; 95% CIs were estimated using 10,000 patient-level bootstrap resamples).

Model	Parameter count (M)	MRE (mm)↓	SDR@2 mm (%)↑	SDR@2.5mm (%)↑	SDR@3 mm (%)↑	SDR@4 mm (%)↑
3D U-Net	19.07	3.8 ± 1.9* 95% CI: 3.2–4.4	48.5 95% CI: 42.5–54.3	62.3 95% CI: 56.3–67.9	73.1 95% CI: 67.7–78.0	85.2 95% CI: 81.3–88.6
V-net	44.03	3.5 ± 1.7* 95% CI: 3.0–4.1	52.8 95% CI: 46.4–59.0	66.7 95% CI: 60.6–72.3	74.4 95% CI: 69.0–79.3	87.9 95% CI: 84.7–90.7
3D HRNet adaptation	22.51	3.2 ± 1.6* 95% CI: 2.7–3.7	58.6 95% CI: 52.4–64.4	71.2 95% CI: 65.6–76.3	80.3 95% CI: 75.6–84.4	89.6 95% CI: 86.6–92.2
H3DE-Net	24.48	2.6 ± 1.5* 95% CI: 2.1–3.1	64.3 95% CI: 58.2–70.0	75.8 95% CI: 70.3–80.7	87.7 95% CI: 83.9–91.0	92.1 95% CI: 89.3–94.4
Proposed method	29.3	2.1 ± 1.2 95% CI: 1.7–2.5	72.0 95% CI: 66.3–77.2	81.8 95% CI: 77.3–85.8	90.3 95% CI: 87.5–92.8	96.7 95% CI: 95.6–97.7

Paired mean MRE, differences (baseline minus proposed) were 1.7 mm (95% CI, 1.3–2.1) for 3D U-Net, 1.4 mm (95% CI, 1.1–1.7) for V-Net, 1.1 mm (95% CI, 0.8–1.4) for the 3D HRNet, adaptation, and 0.5 mm (95% CI, 0.2–0.8) for H3DE-Net. Shapiro–Wilk tests did not indicate departures from normality in the paired differences (W = 0.987, 0.961, 0.973, and 0.982; P = 0.947, 0.234, 0.510, and 0.824, respectively). Holm–Bonferroni-adjusted paired t-test P values were <0.001, <0.001, <0.001, and 0.007, respectively. Wilcoxon sensitivity analyses yielded adjusted P values of <0.001, <0.001, <0.001, and 0.018, respectively, supporting the robustness of the primary comparisons.

At the 2.0 mm threshold, the proposed method achieved an SDR of 72.0%. Errors exceeding this threshold occurred most frequently for the humeral head rotation center, anatomical neck center, and proximal and distal medullary canal centers, which require estimation of internal geometric centers rather than direct identification of discrete surface points. Their mean localization errors ranged from 2.3 to 2.6 mm.

### Per-landmark analysis

Table 6 summarizes landmark-specific localization errors on the test set. The humeral head rotation center and anatomical neck center showed the highest mean errors (both 2.6 mm), followed by the proximal and distal medullary canal centers (2.5 and 2.3 mm, respectively). These four internally defined center landmarks also had relatively low SDR@2.0 mm values, ranging from 47.2% to 55.6%. In contrast, most surface landmarks had mean errors of 1.6–2.1 mm and SDR@2.0 mm values of approximately 80% or higher.

**TABLE 6:** Landmark-specific localization errors on the test set (n = 36); Mean-error 95% CIs were estimated using 10,000 patient-level bootstrap resamples.

Landmark	Mean ± SD (mm)	Median	IQR (mm)	SDR@2.0 mm (%)
HRC	2.6 ± 1.6 95% CI: 2.1–3.1	2.2	1.3–3.2	47.2
ANC	2.6 ± 1.8 95% CI: 2.0–3.2	2.1	1.8–2.8	50.0
ME	2.3 ± 1.1 95% CI: 2.0–2.7	2.1	1.5–2.6	72.2
LE	1.6 ± 0.9 95% CI: 1.3–1.9	1.5	1.1–2.0	83.3
PMC	2.5 ± 1.8 95% CI: 1.9–3.1	1.9	1.2–2.7	52.8
DMC	2.3 ± 1.4 95% CI: 1.8–2.8	1.9	1.1–3.0	55.6
GT	2.1 ± 0.9 95% CI: 1.8–2.4	2.0	1.6–2.5	80.6
GDP	2.0 ± 1.0 95% CI: 1.7–2.3	1.9	1.2–2.7	72.2
SGM	1.9 ± 1.0 95% CI: 1.6–2.3	1.7	1.2–2.4	83.3
TS	1.8 ± 0.8 95% CI: 1.6–2.1	1.7	1.3–2.2	86.1
AI	1.9 ± 0.8 95% CI: 1.7–2.2	1.8	1.4–2.3	83.3
IGM	2.0 ± 0.9 95% CI: 1.7–2.3	1.9	1.5–2.4	80.6
AGM	1.9 ± 0.8 95% CI: 1.7–2.2	1.8	1.3–2.1	80.6
PGM	1.9 ± 0.8 95% CI: 1.7–2.2	1.8	1.6–2.2	80.6

### Qualitative comparison and error distributions

Figure 5 presents representative 3D visualizations of landmark predictions from the proposed method and the baseline models. Larger deviations at the glenoid rim and greater tubercle were observed for 3D U-Net and V-Net, whereas the predictions of the proposed method were generally closer to the ground-truth landmarks. Figure 6 summarizes the landmark-specific error distributions. The humeral head rotation center, anatomical neck center, and proximal and distal medullary canal centers showed wider distributions and more high-error outliers. In contrast, most surface landmarks showed narrower error distributions, consistent with the quantitative results in Table 6.

**FIGURE 5:**
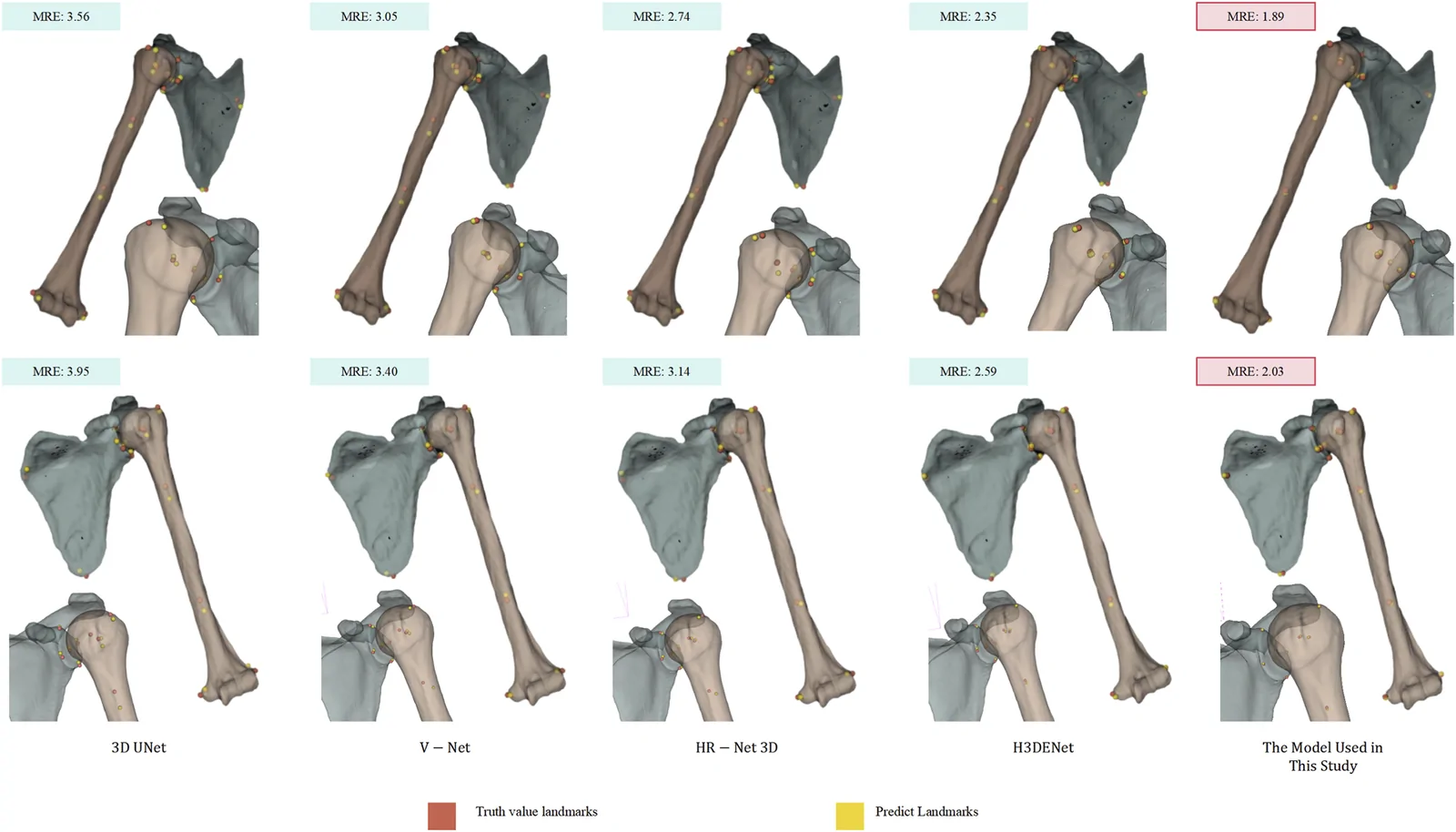
Visual comparison of bony landmark detection across models on 3D reconstructions of representative test cases. Red points: ground truth; yellow points: model predictions.

**FIGURE 6:**
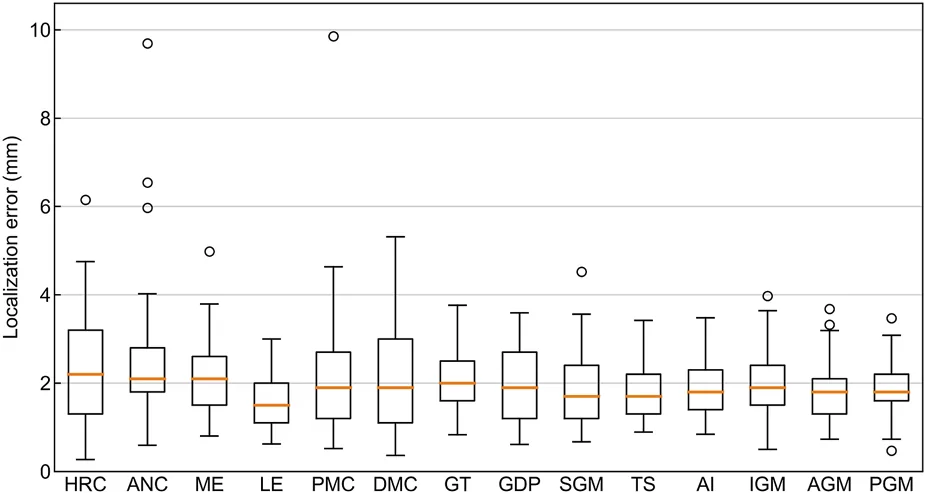
Box-and-whisker plot of the error distribution for 14 bony landmarks on the test set. The box represents the interquartile range; the horizontal line indicates the median; whiskers extend to 1.5×IQR.

### Auxiliary segmentation performance

On the test set, humerus segmentation achieved a Dice coefficient of 0.83 ± 0.05, a 95th-percentile Hausdorff distance (HD95) of 5.8 ± 2.4 mm, and an average symmetric surface distance (ASSD) of 1.2 ± 0.5 mm. The corresponding values for scapula segmentation were 0.78 ± 0.07, 7.6 ± 3.1 mm, and 1.8 ± 0.7 mm, respectively. Representative segmentation results from seven test cases in the sagittal, coronal, and transverse planes are shown in Figure 7.

**FIGURE 7:**
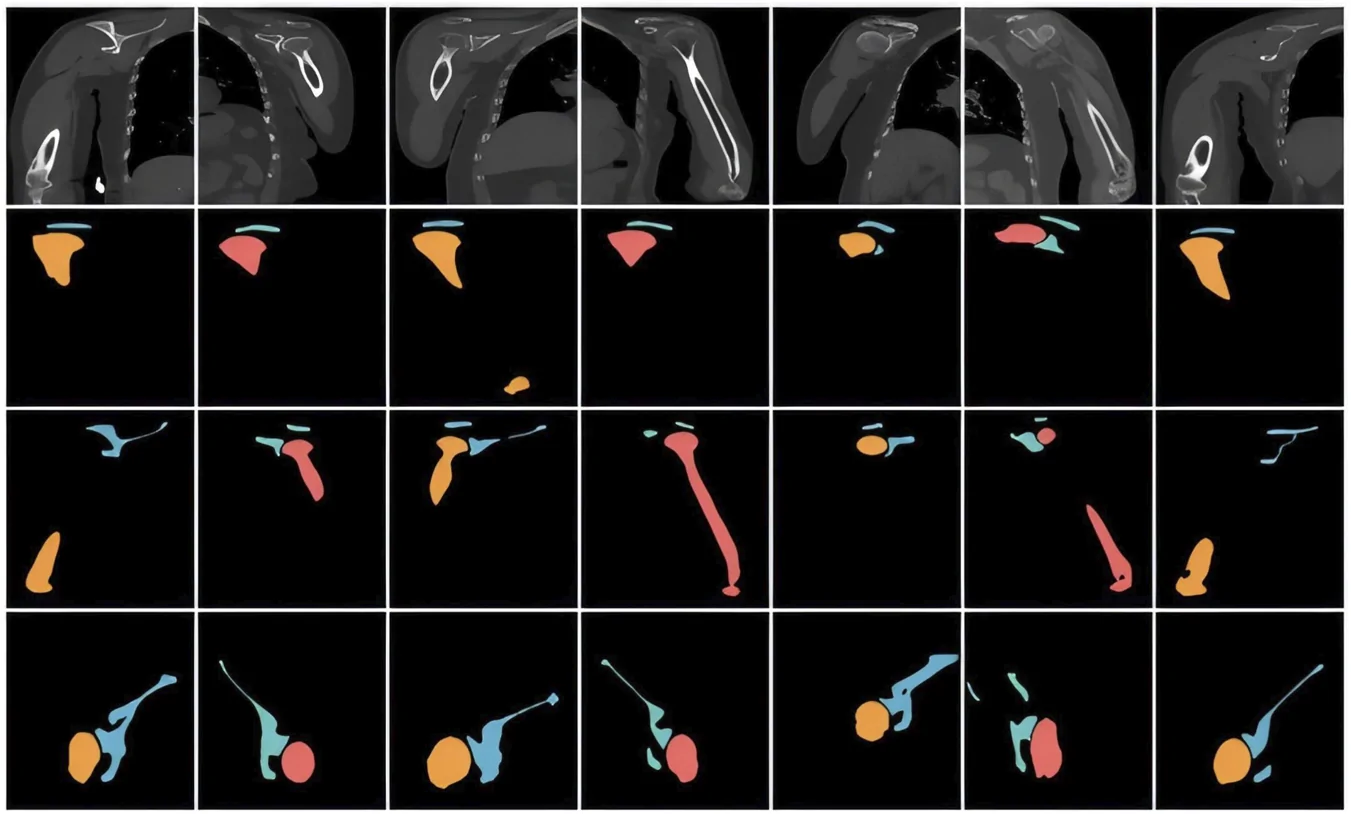
Multi-planar qualitative visualization of auxiliary segmentation predictions on seven test cases. Rows: sagittal, coronal, and transverse planes. Columns: individual test cases.

Figure 8 shows the validation-set training dynamics. The mean Dice coefficient across the humerus and scapula increased and stabilized at approximately 0.83, while the landmark-detection MRE decreased and converged to approximately 2.13 mm. The inverse trend supports the hypothesis that anatomical segmentation serves as a structural anchor during multi-task training.

**FIGURE 8:**
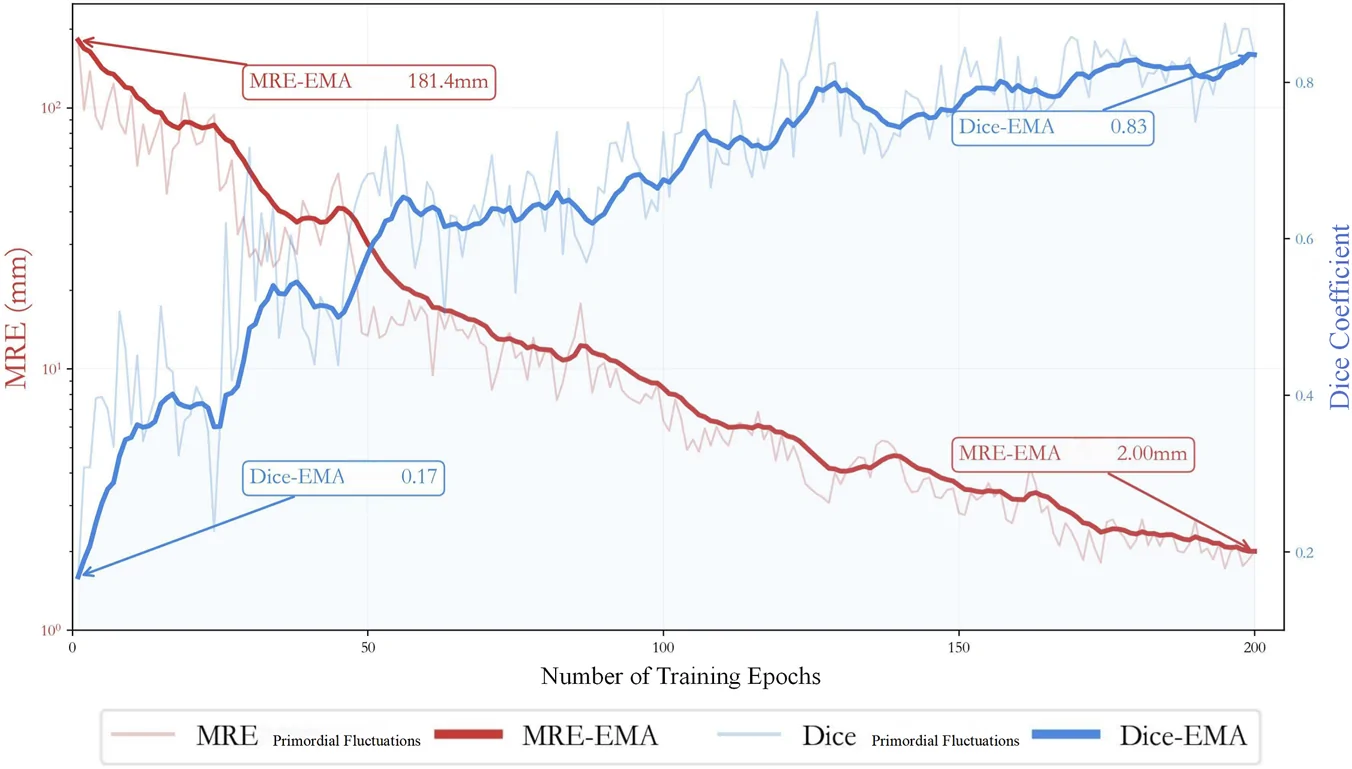
Training dynamics of landmark-detection MRE and the mean segmentation Dice coefficient across the humerus and scapula on the validation set.

### Ablation experiments

Ablation experiments were performed using the same dataset split, preprocessing pipeline, and training settings. Table 7 evaluates the contribution of the main architectural components. Replacing the ResNet-3D encoder with BiFormer reduced the MRE from 3.68 to 3.15 mm and increased SDR@2.0 mm from 50.4% to 59.2%. Adding the auxiliary segmentation decoder with direct feature concatenation further reduced the MRE to 2.74 mm and increased SDR@2.0 mm to 66.5%. Replacing direct concatenation with CtFF produced the best performance, with an MRE of 2.10 mm and an SDR@2.0 mm of 72.0%.

**TABLE 7:** Ablation study of the main model configurations.

Configuration	MRE (mm)	SDR@2.0mm (%)
A: ResNet-3d + single decoder + skip connections	3.68	50.4
B: BiFormer + single decoder + skip connections	3.15	59.2
C: BiFormer + dual decoder + direct concatenation	2.74	66.5
D: BiFormer + dual decoder + CtFF	2.10	72.0

Table 8 evaluates the proposed loss terms using the complete BiFormer–dual-decoder–CtFF architecture. The auxiliary segmentation loss was retained in all configurations and is omitted from the notation for clarity. Adding $L_{geo}$ reduced the MRE by 0.18 mm and increased SDR@2.0 mm by 3.8 percentage points, whereas adding $L_{sim}$ reduced the MRE by 0.10 mm and increased SDR@2.0 mm by 1.7 percentage points. Combining both loss terms produced the largest improvement, reducing the MRE by 0.31 mm and increasing SDR@2.0 mm by 6.7 percentage points.

**TABLE 8:** Ablation of individual loss function components (all with BiFormer + aux. seg. + CtFF). The auxiliary segmentation loss was included and held constant in all configurations and is omitted from the loss notation.

Loss configuration	MRE (mm)	SDR@2.0 mm(%)
$L_{NHM}\text{ only}$	2.41 ± 1.4	65.3
$L_{NHM}+L_{geo}$	2.23 ± 1.3	69.1
$L_{NHM}+L_{sim}$	2.31 ± 1.4	67.0
$L_{NHM}+L_{geo}+L_{sim}\;\left(\text{Ours}\right)$	2.10 ± 1.2	72.0

### Computational efficiency

Table 9 summarizes the model size and inference efficiency of each method. The proposed model contained 29.3 million parameters and required 24.7 ± 1.6 s per volume, with a peak GPU memory consumption of 8.6 GB. It was slightly slower than 3D HRNet adaptation but 10.4 s faster than H3DE-Net while using 0.6 GB less GPU memory. Although computationally more demanding than 3D U-Net and V-Net, its mean inference time of approximately 25 s per volume remains compatible with offline preoperative planning.

**TABLE 9:** Computational efficiency on test volumes. Inference time and peak GPU memory were measured on a single NVIDIA RTX 4090 GPU using an input size of 192 × 192 × 192.

Model	Parameters (M)	Inference time (s/volume)	GPU memory (GB)
3D U-Net	19.07	8.2 ± 0.5	4.1
V-net	44.03	15.6 ± 1.2	8.3
3D HRNet adaptation	22.51	22.4 ± 1.8	7.8
H3DE-net	24.48	35.1 ± 2.3	9.2
Ours	29.3	24.7 ± 1.6	8.6

### Failure case analysis

The five test cases with the highest per-case MREs (3.4–4.1 mm) were examined to identify potential failure patterns. Larger deviations were observed in cases with thin scapular bodies or pronounced variations in scapular curvature, particularly for the trigonum spinae and glenoid-rim landmarks. Marked humeral abduction or external rotation was also associated with larger errors in the humeral head rotation center and medullary canal centers. These observations suggest that localization performance may be affected by anatomical variation and arm positioning.

At the landmark level, the humeral head rotation center and anatomical neck center had the highest mean errors (both 2.6 mm), and the humeral head rotation center had the lowest SDR@2.0 mm (47.2%). The proximal and distal medullary canal centers also showed relatively high errors of 2.5 and 2.3 mm, respectively. These landmarks depend on estimation of internal geometric centers rather than direct identification of discrete surface points.

## Discussion

The main findings of this study are as follows: On intact contralateral shoulder CT, which provides reference anatomy for mirror-based preoperative planning, the proposed multi-task dual-decoder framework achieved lower MRE and higher SDR than the baseline networks. Architecture-ablation experiments showed progressive improvements after introducing the BiFormer encoder, auxiliary segmentation decoder, and CtFF module. The BiFormer region-routing mechanism identifies anatomically relevant regions before applying fine-grained attention, enabling efficient modeling of long-range spatial relationships between the humerus and scapula. Meanwhile, residual depthwise convolutions preserve local features such as cortical boundaries and bony prominences, while sparse routing limits computation to relevant regions. This combination of global anatomical context and local surface information may explain the improved localization of glenoid-rim and other anatomically complex landmarks.

The multi-task design provided an additional source of performance improvement. On the test set, the auxiliary segmentation branch achieved Dice coefficients of 0.83 for the humerus and 0.78 for the scapula. The lower scapular performance may be related to its thinner and more complex morphology. The segmentation decoder uses voxel-level humeral and scapular contours as auxiliary supervision, while CtFF transfers bone-structural information to the landmark decoder and reduces interference from surrounding non-bony structures. Architecture-ablation experiments showed progressive improvements from the single-decoder model to the dual-decoder model and subsequently to the CtFF-enhanced model. These findings suggest that auxiliary segmentation provides useful structural guidance rather than serving as the final clinical output. The proposed framework is therefore intended as an upstream localization component for subsequent mirror registration and reconstruction.

The composite loss function also played a measurable role. The geometric structure constraint loss reduced errors for landmark pairs with well-established anatomical distances, while the distance-aware similarity learning loss improved feature discriminability at the voxel level. The ablation results in Table 8 showed that each term contributed independently and that their combination produced the largest improvement.

It is important to note that the evaluation cohort in this study consisted exclusively of images of the intact contralateral shoulder. The results should not be extrapolated to suggest that the model performs equally well on shoulders with bone defects, malunion, severe osteophytes, or significant glenoid wear. The current work is best positioned as a validation of an upstream module in the mirror-based preoperative planning workflow—first localizing the anatomical reference on the healthy side, then combining this with mirror registration to reconstruct the affected side.

The SDR@2.0 mm of 72.0% indicates that most, but not all, landmarks met the predefined accuracy threshold. Errors were concentrated in internally defined center landmarks, particularly the humeral head rotation center, anatomical neck center, and medullary canal centers. Unlike discrete surface landmarks, these points require estimation from global bone morphology or canal geometry. Their lower accuracy indicates that further improvement is required before the model can be incorporated into clinical planning workflows.

The current workflow requires CT imaging of the intact contralateral shoulder, which increases radiation exposure. Compared with CT, ultrasound does not involve ionizing radiation and provides real-time imaging; it is also widely available and relatively inexpensive. These characteristics are relevant to robot-assisted shoulder arthroplasty because mirror-based preoperative planning currently relies on CT of the intact contralateral shoulder to obtain three-dimensional landmarks and bone-surface models, which are then mirrored or registered to the affected side to guide implant positioning. Additional CT imaging of the healthy shoulder increases cumulative radiation exposure. Although low image quality and a limited field of view remain challenges for ultrasound-based three-dimensional bone modelling (Hohlmann et al., 2024), recent studies provide a technical basis for further investigation. UltraBones100 k generated automated bone labels by superimposing tracked CT bone models on tracked ultrasound images and released 100,000 *ex vivo* images of the fibula, tibia, and foot (Wu et al., 2025). NeuralBoneReg further evaluated self-supervised multimodal bone-surface registration using CT–ultrasound datasets of the fibula, tibia, femur, and pelvis (Wu et al., 2026). Future studies could therefore examine whether three-dimensional ultrasound of the intact shoulder can replace additional contralateral CT acquisition. Ultrasound-derived landmarks and bone-surface models could then be mirrored or registered to the affected side to support robot-assisted implant planning while reducing radiation exposure.

This study has several limitations. It was a single-center retrospective study involving 179 shoulders, and larger multicenter cohorts are needed to assess generalizability across scanners and imaging protocols. Evaluation was restricted to anatomically intact contralateral shoulders; performance on shoulders with bone defects, malunion, severe osteophytes, or glenoid wear remains unknown. Although consensus review reduced inter-observer variability, manual annotation remains a potential source of subjective error. Finally, the model was evaluated only as an upstream localization component rather than as part of a complete robotic planning system. Although real-world auto-planning has been shown to reduce planning time and user interactions (Mahe et al., 2026), prospective integration studies are still needed to determine whether incorporating the present method improves implant-positioning accuracy, end-to-end workflow efficiency, or patient outcomes.

In summary, this study developed a multi-task 3D bony landmark detection network designed to automatically acquire anatomical reference points from the unaffected side for preoperative planning of total shoulder arthroplasty. The network achieved superior numerical results on this dataset and provides a methodological foundation for subsequent automated measurement of clinical parameters and automated planning.

## Data Availability

The original contributions presented in the study are included in the article/supplementary material, further inquiries can be directed to the corresponding author.
